# Psychological and socioeconomic impact of long-term home management on patients with left ventricular assist devices and their caregivers: a nationwide multicenter questionnaire survey

**DOI:** 10.1007/s10047-026-01569-4

**Published:** 2026-07-09

**Authors:** Michiko Watanabe, Goro Matsumiya, Hiroki Kohno, Koichiro Kinugawa

**Affiliations:** 1 Department of Cardiovascular Surgery, Chiba University Hospital, Chiba, Japan; 2 Second Department of Internal Medicine, Toyama University, Toyama, Japan

**Keywords:** LVAD, Caregiver burden, Mechanical circulatory support, Quality of life, Home care

## Abstract

Left ventricular assist device (LVAD) therapy requires safe long-term home management supported by patients, caregivers, and healthcare systems. In Japan, continuous caregiver availability was historically required or strongly expected in LVAD home management, although this framework has recently been relaxed. We evaluated the psychological and socioeconomic burden experienced by LVAD patients and primary caregivers under this former 24-h caregiver-accompaniment framework. This cross-sectional, nationwide anonymous web-based questionnaire survey was conducted through 79 LVAD centers between April and July 2024. De-identified individual-level data from 184 patients and 136 primary caregivers were analyzed. Prespecified comparisons of depressive symptoms according to caregiver-accompaniment burden and work/study status were performed within each respondent group, and multivariable logistic regression was used to identify factors associated with depressive symptoms. Psychological burden related to caregiver accompaniment was reported by 83.7% of patients and 61.8% of caregivers. Not working or studying was reported by 44.0% and 31.6%, respectively. Depressive symptoms were reported in 36.4% of patients and 35.3% of caregivers. Depressive symptoms were more frequent among respondents with caregiver-accompaniment burden than among those without such burden in both patients (61/154 [39.6%] vs. 6/30 [20.0%]; *p* = 0.041) and caregivers (38/84 [45.2%] vs. 10/52 [19.2%]; *p* = 0.002), whereas depressive symptoms did not differ significantly according to work/study status. Under the former 24-h caregiver-accompaniment framework, Japanese LVAD households reported substantial psychological and socioeconomic burdens. These findings provide real-world baseline data for evaluating recent policy relaxation and for designing support systems that promote quality of life and social reintegration while maintaining safety.

**Supplementary Information:**

The online version contains supplementary material available at https://doi.org/10.1007/s10047-026-01569-4.

## Introduction

Left ventricular assist devices (LVADs) have become an established treatment option for patients with advanced heart failure, with substantial improvements in survival and clinical outcomes over the past decade [1–3]. The evolution of continuous-flow technology and the accumulation of large registry data have expanded the role of LVAD therapy in end-stage heart failure management [1–3].

In Japan, LVAD therapy has developed within a highly regulated clinical framework. Nationwide registry data from the Japanese Registry for Mechanically Assisted Circulatory Support have documented patient characteristics, device profiles, survival, and adverse events in real-world Japanese practice [4]. National guidance has also defined the institutional and clinical framework for implantable LVAD therapy, reflecting the structured nature of advanced heart failure care in Japan [5]. More recently, Japanese reports have highlighted changing concepts of home LVAD management, caregiver shortages, and the need to reduce caregiver burden as technology and healthcare-system support evolve [6, 7].

Despite these advances, long-term LVAD home management continues to place practical, psychological, and socioeconomic demands on both patients and family caregivers. Previous studies have described caregiver burden, reduced quality of life, and psychosocial distress in LVAD households [8–13]. The former Japanese framework, in which 24-h caregiver accompaniment was historically required or strongly expected in practice, provides a unique context in which to examine whether home-management structures affect work, study, social participation, and mental well-being at the household level. This nationwide, multicenter questionnaire survey therefore aimed to clarify the psychological and socioeconomic burden experienced by LVAD patients and primary caregivers under the former 24-h caregiver-accompaniment framework in Japan.

**Fig. 1:**
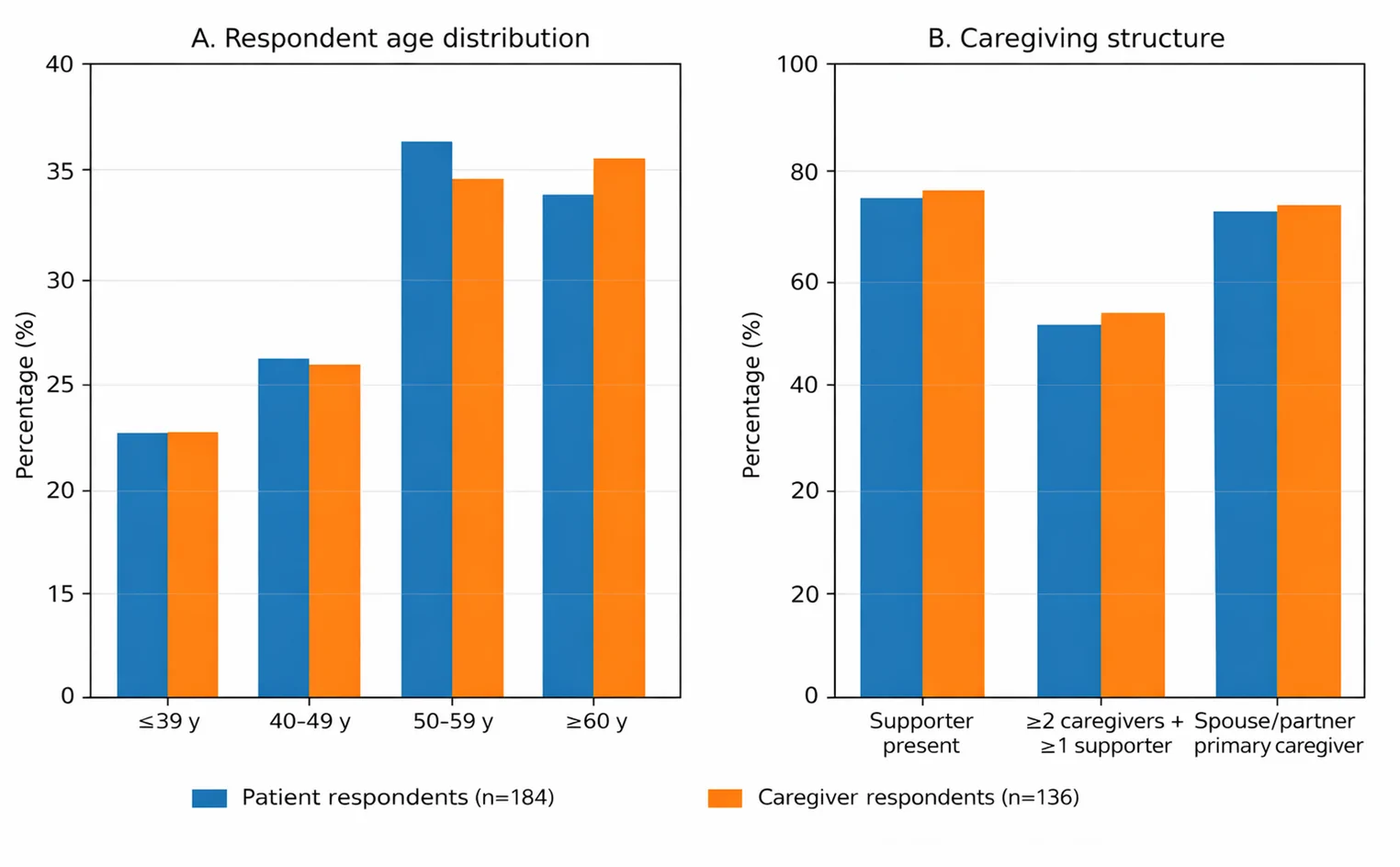
Respondent characteristics and caregiving structure. Panel A shows respondent age distribution in patients and caregivers. Panel B shows caregiving-structure variables derived separately from patient reports and caregiver reports: supporter presence, the most common arrangement of two or more caregivers plus at least one supporter, and spouse/partner as the primary caregiver

**Fig. 2:**
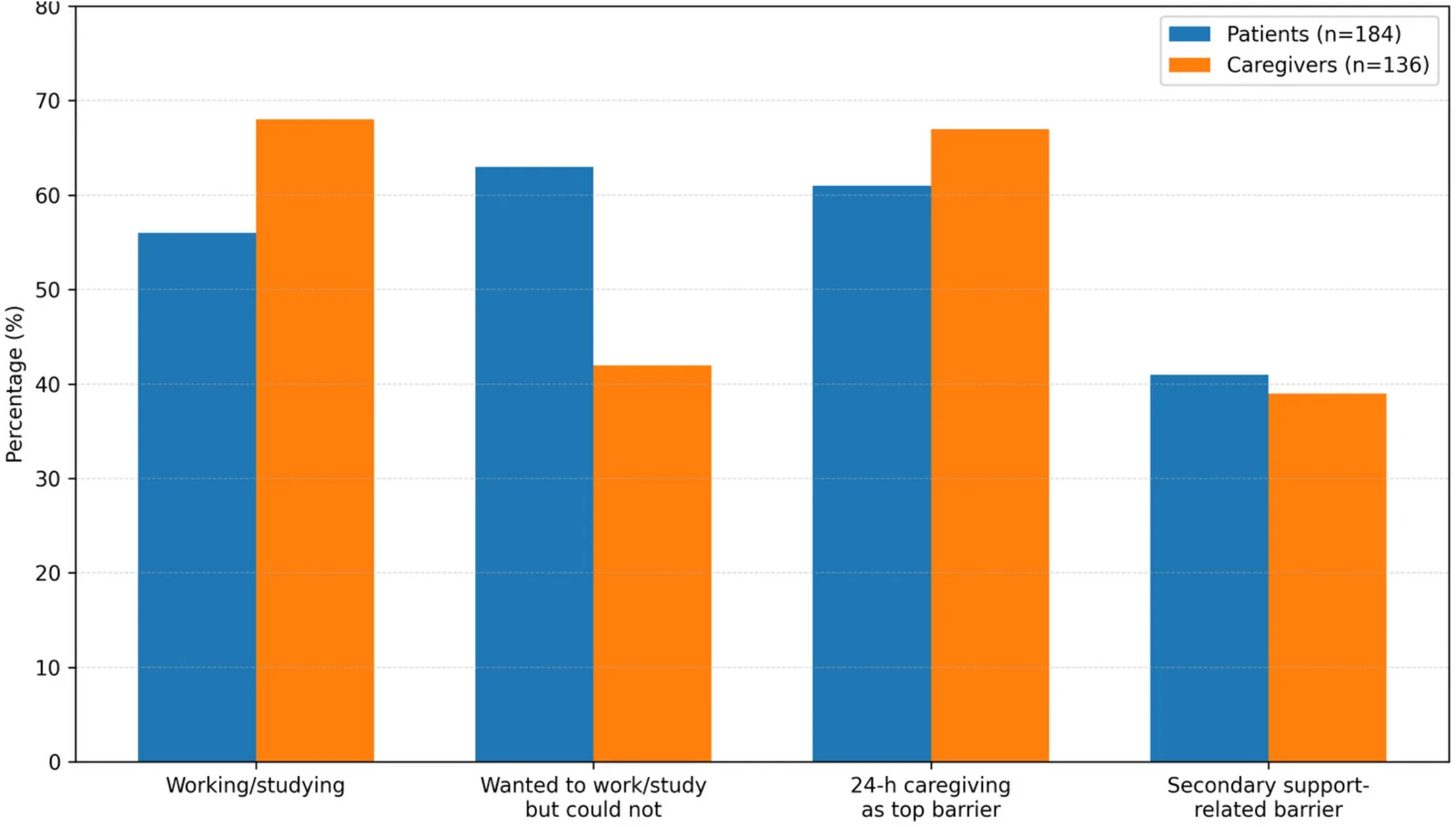
Employment and study restrictions related to caregiver accompaniment. The figure summarizes work/study status, inability to work or study despite willingness, and the major reported barriers among patients and caregivers. Percentages for wanting to work or study but being unable to do so are calculated among respondents who were not working or studying

**Fig. 3:**
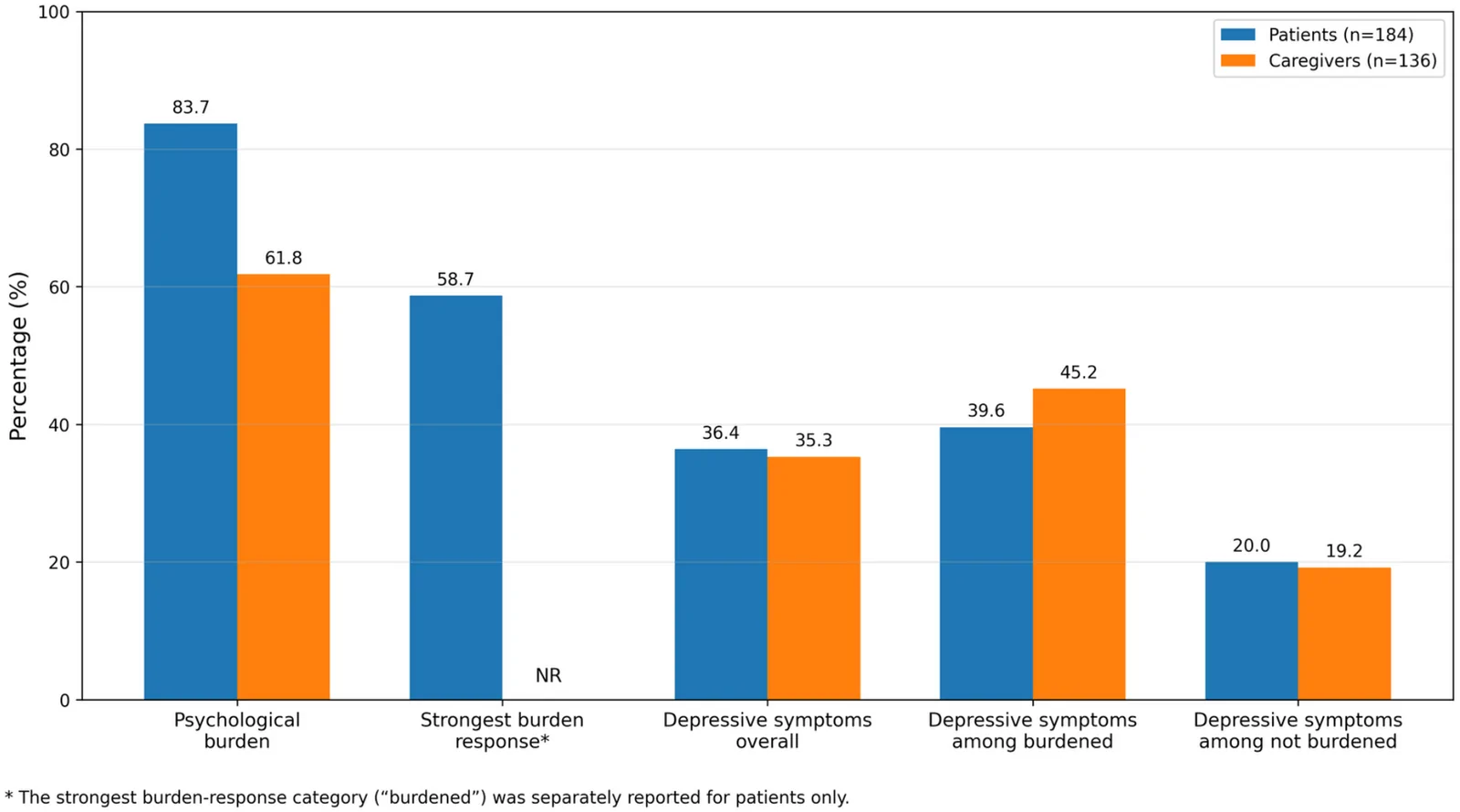
Psychological burden, depressive symptoms, and multivariable analysis. The figure shows overall caregiver-accompaniment burden, depressive symptoms stratified by burden status, and odds ratios for factors associated with PHQ-2 positivity in multivariable logistic regression. CI, confidence interval; OR, odds ratio; PHQ-2, two-item Patient Health Questionnaire

## Materials and methods

### Study design and participants

This study was designed as a cross-sectional, nationwide, anonymous web-based questionnaire survey involving LVAD centers across Japan. The survey was conducted between April 2 and July 31, 2024. Survey information and a QR code were provided through physicians at 79 participating LVAD centers, who then invited eligible patients and primary caregivers to respond. Eligible patient respondents were adults aged 18 years or older who were receiving ventricular assist device support and had undergone implantation at least 6 months earlier; their primary caregivers were also eligible. A total of 320 responses were obtained, comprising 184 patients and 136 primary caregivers.

The dataset available for analysis consisted of de-identified individual-level responses. However, to preserve anonymity, the dataset did not include center identifiers, the number of eligible individuals invited at each center, or information on nonrespondents. Therefore, the overall response rate, center-level response rates, and comparisons between respondents and nonrespondents could not be calculated.

### Questionnaire and variables

The questionnaire contained 27 items and assessed household caregiving structure, including the number of caregivers and supporters, employment or study status, psychological burden from caregiver accompaniment, depressive symptoms, daily living abilities, use of home services, device-handling ability, device-related emergency experience, perceived benefits after implantation, and desired future changes. Detailed questionnaire-level descriptive data are provided in Supplementary Tables S1–S4. A supporter was defined as an individual other than a caregiver who had received designated training and could be asked to provide emergency assistance, mainly in the event of VAD pump stoppage. Supporters were therefore distinct from primary caregivers and were not necessarily involved in daily caregiving.

Unless otherwise specified, responses reflected the respondent’s status at the time of questionnaire completion. Depressive symptoms were assessed using the two-item Patient Health Questionnaire (PHQ-2), and PHQ-2 positivity was defined as a total score of 3 or higher. The questionnaire included duration since LVAD implantation and time from implantation to first discharge, but it did not directly record the interval from first discharge to questionnaire completion. For the item assessing burden related to 24-h caregiver accompaniment, respondents in the destination-therapy context were asked to recall the first 6 months after discharge.

### Statistical analysis

Continuous variables are presented as mean ± standard deviation or median and interquartile range, as appropriate, and categorical variables are presented as frequencies and percentages. LVAD support duration was calculated from questionnaire-based categories using category midpoints; for the category of 6 years or longer, the numeric free-text response was used when available. Inferential testing for the primary reviewer-requested comparisons was limited to prespecified 2 × 2 comparisons of depressive symptoms according to caregiver-accompaniment burden and work/study status within each respondent group. Pearson’s chi-squared test was used for these comparisons; Fisher’s exact test was planned when expected cell counts were less than 5. Prevalence differences with 95% confidence intervals were calculated and expressed in percentage points. Patient-versus-caregiver differences in descriptive questionnaire responses were not treated as primary inferential comparisons. LVAD support duration was additionally stratified into less than 1 year, 1 to less than 3 years, and 3 years or longer for exploratory sensitivity analyses.

Multivariable logistic regression analysis was performed separately for patients and caregivers to identify factors associated with depressive symptoms. Variables included caregiver-accompaniment burden, work/study status, LVAD support duration, and the number of caregivers. A two-sided p value of less than 0.05 was considered statistically significant. Statistical analyses were performed using Python 3.11 with SciPy and statsmodels. This study was approved by the ethics committee of Chiba University Hospital (HK202312-04) and was conducted in accordance with the Declaration of Helsinki. Informed consent was obtained from all participants before enrollment.

## Results

### Study population and caregiving structure

Baseline characteristics and caregiving structure are summarized in Table 1 and Fig. 1. The patient group comprised 184 LVAD recipients, and the caregiver group comprised 136 primary caregivers. Male sex was more frequent among patients than among caregivers (87.5% vs. 58.1%). The mean LVAD support duration was 3.40 ± 1.72 years in patient reports and 3.19 ± 1.69 years in caregiver reports; the corresponding medians were 3.5 years and 3.0 years, with interquartile ranges of 1.5 to 4.5 years in both groups. The full LVAD support-duration distribution is provided in Supplementary Table S5.

**Table 1:** Baseline characteristics and caregiving structure of the study participants

Variable	Patients (n = 184)	Caregivers (n = 136)
*Age group, years, n (%)*		
< = 39	31 (16.8)	23 (16.9)
40–49	36 (19.6)	26 (19.1)
50–59	61 (33.2)	43 (31.6)
> = 60	56 (30.4)	44 (32.4)
Male sex, n (%)	161 (87.5)	79 (58.1)
LVAD support duration, years, mean ± SD	3.40 ± 1.72	3.19 ± 1.69*
LVAD support duration, years, median (IQR)	3.5 (1.5–4.5)	3.0 (1.5–4.5)*
LVAD support duration, years, range	0.75–9.0	0.75–8.0*
*Time from implantation to first discharge, n (%)*		
< 3 months	94 (51.1)	68 (50.0)
3 to < 6 months	71 (38.6)	52 (38.2)
> = 6 months	19 (10.3)	16 (11.8)
*Treatment goal, n (%)*		
Bridge to transplant	156 (84.8)	112 (82.4)*
Destination therapy	24 (13.0)	22 (16.2)*
Unknown	4 (2.2)	2 (1.5)*
Number of caregivers per patient, mean ± SD	2.62 ± 1.31†	2.55 ± 1.22
Supporter present, n (%)	139 (75.5)	105 (77.2)
Number of supporters, mean ± SD	2.32 ± 1.86	2.30 ± 1.74
> = 2 caregivers plus > = 1 supporter, n (%)	104 (56.5)	80 (58.8)
Primary caregiver spouse/partner, n (%)	135 (73.4)	102 (75.0)
Primary caregiver other than spouse/partner, n (%)	49 (26.6)	34 (25.0)

*Values reported by caregivers refer to the LVAD patient being cared for when applicable. † One patient with an unknown number of caregivers was excluded from the mean calculation. IQR, interquartile range; LVAD, left ventricular assist device; SD, standard deviation

Caregiving networks were common. The mean number of caregivers per patient was 2.62 according to patient reports and 2.55 according to caregiver reports. Supporters were present in 75.5% and 77.2% of households, respectively, and the most common arrangement was two or more caregivers plus at least one supporter (56.5% and 58.8%). Primary caregivers were most frequently spouses or partners, accounting for 73.4% of patient reports and 75.0% of caregiver reports. Detailed caregiving-structure and support-network data are provided in Supplementary Table S1.

### Employment, study, and caregiver-accompaniment burden

Major questionnaire outcomes are summarized in Table 2. Restrictions on employment and study, shown in Fig. 2 were frequent in both groups. Not working or studying was reported by 44.0% of patients and 31.6% of caregivers. Among respondents who were not working or studying, 63.0% of patients and 41.9% of caregivers wished to work or study but could not. Among those unable to work or study despite willingness, the top reported barrier was difficulty obtaining 24-h accompaniment from the main caregiver among patients (60.8%) and the need to accompany the patient 24 h a day among caregivers (66.7%). Secondary barriers included lack of support from family members other than the main caregiver among patients (41.2%) and difficulty rotating care with other caregivers among caregivers (38.9%). Detailed work/study responses and reasons for inability to work or study are provided in Supplementary Table S2.

**Table 2:** Major questionnaire outcomes and prespecified comparisons of depressive symptoms according to caregiver-accompaniment burden and work/study status

Outcome / comparison	Patients (n = 184)	Caregivers (n = 136)	Prespecified statistical result
Working or studying, n (%)	103 (56.0)	93 (68.4)	Descriptive only
Not working or studying, n (%)	81 (44.0)	43 (31.6)	Descriptive only
Among those not working/studying, wanted to work or study but could not, n/N (%)	51/81 (63.0)	18/43 (41.9)	Descriptive only
Top barrier among those unable to work/study despite willingness, n/N (%)	31/51 (60.8)‡	12/18 (66.7)§	Descriptive only
Secondary barrier related to support structure, n/N (%)	21/51 (41.2)||	7/18 (38.9)¶	Descriptive only
Psychological burden related to caregiver accompaniment, n (%)	154 (83.7)	84 (61.8)	Descriptive only
Strongest burden-response category (“burdened”), n (%)	108 (58.7)	38 (27.9)	Descriptive only
Depressive symptoms in the past 2 weeks, n (%)	67 (36.4)	48 (35.3)	Descriptive only
Depressive symptoms by caregiver-accompaniment burden: with burden vs. without burden	61/154 (39.6) vs. 6/30 (20.0)	38/84 (45.2) vs. 10/52 (19.2)	Patients: prevalence difference 19.6 (95% CI, 0.8 to 32.7), * p* = 0.041; caregivers: prevalence difference 26.0 (95% CI, 9.7 to 39.6), * p* = 0.002
Depressive symptoms by work/study status: not working/studying vs. working/studying	34/81 (42.0) vs. 33/103 (32.0)	16/43 (37.2) vs. 32/93 (34.4)	Patients: prevalence difference 9.9 (95% CI, -4.0 to 23.6), * p* = 0.164; caregivers: prevalence difference 2.8 (95% CI, -13.5 to 20.2), * p* = 0.751
Can handle the VAD device appropriately, n (%)	85 (46.2)	51 (37.5)	Descriptive only
Any positive response indicating VAD handling was possible, n (%)	171 (92.9)	126 (92.6)	Descriptive only
Experienced a VAD-related emergency, n (%)	31 (16.8)	24 (17.6)	Descriptive only

Inferential results are shown only for the prespecified 2 × 2 comparisons of depressive symptoms according to caregiver-accompaniment burden and work/study status within each respondent group. Prevalence differences are expressed in percentage points. Patient-versus-caregiver differences in descriptive questionnaire responses were not treated as primary inferential comparisons. ‡ Difficulty obtaining 24-h accompaniment from the main caregiver. § Need to accompany the patient 24 h a day. || Lack of support from family members other than the main caregiver. ¶ Difficulty rotating care with other caregivers. CI, confidence interval; PHQ-2, two-item Patient Health Questionnaire; VAD, ventricular assist device

Psychological burden related to the former 24-h caregiver-accompaniment framework was reported by 154 patients (83.7%) and 84 caregivers (61.8%)(Fig. 3). The strongest burden-response category, 'burdened,' was selected by 108 patients (58.7%) and 38 caregivers (27.9%). Detailed burden-response categories and PHQ-2 depressive-symptom responses are provided in Supplementary Table S3. Free-text responses indicated that patients frequently experienced guilt about restricting family members’ freedom, imposing practical burden, and being unable to go out alone. Caregivers commonly described persistent vigilance, restricted freedom, limited private time, and work restrictions.

### Depressive symptoms and additional analyses

Depressive symptoms during the 2 weeks before questionnaire completion were reported by 67 patients (36.4%) and 48 caregivers (35.3%). In prespecified comparisons, depressive symptoms were significantly more frequent among respondents who reported caregiver-accompaniment burden than among those who did not, both in patients [61/154 (39.6%) vs. 6/30 (20.0%); prevalence difference, 19.6 percentage points; 95% CI 0.8–32.7; *p* = 0.041] and caregivers [38/84 (45.2%) vs. 10/52 (19.2%); prevalence difference, 26.0 percentage points; 95% CI 9.7–39.6; *p* = 0.002]. In contrast, depressive symptoms did not differ significantly according to work/study status in either patients [not working/studying vs. working/studying: 34/81 (42.0%) vs. 33/103 (32.0%); prevalence difference, 9.9 percentage points; 95% CI, -4.0 to 23.6; *p* = 0.164] or caregivers [16/43 (37.2%) vs. 32/93 (34.4%); prevalence difference, 2.8 percentage points; 95% CI, − 13.5 to 20.2; *p* = 0.751].

In multivariable logistic regression analysis (Supplementary Table S8), caregiver-accompaniment burden was associated with depressive symptoms among caregivers (odds ratio [OR], 3.42; 95% confidence interval [CI], 1.51–7.76; *p* = 0.003). Among patients, the association between caregiver-accompaniment burden and depressive symptoms did not reach conventional statistical significance (OR, 2.61; 95% CI, 0.99–6.86; *p* = 0.053). Work/study status, LVAD support duration, and the number of caregivers were not independently associated with depressive symptoms in either group.

Exploratory analyses stratified by LVAD support duration and by treatment goal are shown in Supplementary Tables S6 and S7, respectively. Psychological burden remained frequent across LVAD duration strata. In patient respondents, PHQ-2 positivity differed across duration strata (< 1 year, 11.1%; 1 to < 3 years, 47.9%; >  = 3 years, 30.4%; *p* = 0.016), although the < 1-year stratum was small. In BTT/DT subgroup analyses, DT caregivers showed a numerically higher prevalence of depressive symptoms than BTT caregivers (54.5% vs. 32.1%; *p* = 0.045), but the DT subgroup was small and these findings should be interpreted as exploratory.

In device-management items, ‘can handle the VAD device appropriately’ was selected by 46.2% of patients and 37.5% of caregivers, whereas 92.9% and 92.6%, respectively, provided some positive response indicating that device handling was possible. A prior VAD-related emergency was reported by 16.8% of patients and 17.6% of caregivers. Additional daily living, home-service use, VAD-handling, and emergency-experience results are summarized in Supplementary Table S4.

## Discussion

This nationwide, multicenter survey demonstrated that LVAD patients and primary caregivers in Japan experienced substantial psychological and socioeconomic burdens under the former 24-h caregiver-accompaniment framework. The main findings were as follows. First, psychological burden related to caregiver accompaniment was highly prevalent, particularly among patients. Second, work and study restrictions were common in both patients and caregivers. Third, depressive symptoms were more frequent among respondents who reported caregiver-accompaniment burden, and multivariable analysis showed a significant association between burden and depressive symptoms among caregivers. These findings provide quantitative evidence that the consequences of LVAD home management extend beyond device handling and clinical safety to affect social participation, employment, education, and mental well-being.

A key interpretative point is that this study captured experiences under a former Japanese LVAD home-management framework in which continuous caregiver availability was historically required or strongly expected in practice. This framework has recently been relaxed, and current LVAD care increasingly emphasizes healthcare-system support and flexible home management. Therefore, the present findings should not be interpreted as a description of current formal requirements alone. Rather, they provide real-world baseline data on household-level burden generated by prolonged caregiver accompaniment and may help evaluate whether recent policy relaxation reduces psychological and socioeconomic burden while maintaining patient safety.

Recent Japanese reports by Saito et al. provide an important system-level context for interpreting the present findings. Saito et al. discussed evolving paradigms in home LVAD management and the need to reduce caregiver burden in the era of advanced technology, and our findings provide real-world patient- and caregiver-reported baseline data on psychological burden, employment restrictions, and depressive symptoms against which the impact of such changes in home-management frameworks can be evaluated [6]. In addition, Saito et al. identified caregiver shortage as the foremost barrier to the expansion of treatment for end-stage heart failure in Japan, whereas the present survey quantitatively demonstrates the household-level psychological and socioeconomic burden experienced by patients and caregivers that may underlie this shortage [7]. Taken together, these data suggest that future LVAD support systems should be assessed not only by clinical outcomes but also by their ability to reduce household-level burden and facilitate social reintegration of both patients and caregivers.

The high prevalence of burden despite frequent presence of multiple caregivers and supporters suggests that support structure alone may not eliminate household-level strain when the overall framework requires constant availability and vigilance.

The relationship between caregiver-accompaniment burden and depressive symptoms is clinically important. In prespecified unadjusted comparisons, depressive symptoms were significantly more frequent among both patients and caregivers who reported caregiver-accompaniment burden. In multivariable analysis, this association remained particularly clear among caregivers, whereas the corresponding association among patients was attenuated after adjustment. This finding is consistent with broader caregiving literature showing that sustained family caregiving may adversely affect mental and physical health [9, 11]. It also supports the view that psychosocial assessment in LVAD care should include caregivers as integral members of the treatment unit, rather than focusing only on patient-level clinical outcomes [12, 13].

The socioeconomic implications are also important. Not working or studying was reported by 44.0% of patients and 31.6% of caregivers, and a substantial proportion of these respondents wished to work or study but could not. The top barriers directly reflected caregiver accompaniment and rotation difficulties. These findings suggest that safety-oriented LVAD home-care systems should be balanced with support models that protect caregivers’ autonomy, employment, and mental health while supporting patient reintegration into society.

The exploratory subgroup analyses provide additional context (Supplementary Tables S6 and S7). Burden remained frequent across LVAD duration strata, indicating that caregiver-accompaniment burden is not limited to the early post-discharge period. However, depressive symptoms varied across duration strata among patients, and DT caregivers showed a numerically higher prevalence of depressive symptoms than BTT caregivers. These findings should be interpreted cautiously because several subgroups were small, but they suggest that support needs may differ according to time since implantation and treatment goal. Future studies with larger DT samples and detailed longitudinal follow-up from discharge are needed.

Remote management and healthcare-system-based backup support may offer one possible direction for reducing household-level burden. However, evidence from non-LVAD heart failure populations, such as the TIM-HF2 trial, cannot be directly extrapolated to LVAD households because LVAD management includes device-specific emergency response, driveline care, power-source management, and caregiver training [14]. Any remote or flexible support model for LVAD patients should therefore be evaluated specifically for LVAD safety, emergency response, caregiver burden, and patient quality of life.

This study has several limitations. First, although de-identified individual-level data were available for statistical analysis, the dataset did not include center identifiers, the number of eligible individuals invited at each center, or information on nonrespondents. Therefore, center-level response rates and comparisons between respondents and nonrespondents could not be calculated, and selection bias cannot be excluded. Second, the interval from first discharge to questionnaire completion was not directly recorded. Although LVAD support duration and the interval from implantation to first discharge were captured, respondents may have been at different stages of adaptation to home management. Third, the DT subgroup was small; therefore, BTT/DT subgroup findings should be interpreted as exploratory (Supplementary Table S7). Fourth, because of the cross-sectional design, causal relationships between caregiver-accompaniment burden, employment or study restrictions, and depressive symptoms cannot be inferred. Finally, the study reflects the Japanese LVAD home-care context and may not be fully generalizable to countries with different care systems. Nevertheless, the nationwide multicenter design, inclusion of both patient and caregiver perspectives, availability of individual-level data, and supplementary questionnaire-level data (Supplementary Tables S1-S8) provide a broad view of the household-level burden associated with long-term LVAD home management in Japan.

## Conclusion

Under the former 24-h caregiver-accompaniment framework, Japanese LVAD households reported substantial psychological and socioeconomic burdens, including restrictions on work or study and frequent depressive symptoms. Caregiver-accompaniment burden was associated with depressive symptoms, particularly among caregivers. These findings provide real-world baseline data for evaluating recent policy relaxation and for designing more flexible support systems that maintain patient safety while promoting quality of life, caregiver autonomy, and social reintegration.

## Supplementary Information

Below is the link to the electronic supplementary material.

Supplementary Material 1

Supplementary Material 1

Supplementary Material 2

Supplementary Material 3

## Data Availability

The data supporting the findings of this study are available within the article and its supplementary materials. Additional individual-level data are not publicly available due to privacy and ethical restrictions.
