# Supplementary material for: Psychological and socioeconomic impact of long-term home management on patients with left ventricular assist devices and their caregivers: a nationwide multicenter questionnaire survey
Source: J Artif Organs. 2026 Jul 9;29(3):43. doi: 10.1007/s10047-026-01569-4 (PMC13350110; doi:10.1007/s10047-026-01569-4)
Supplement: Supplementary file 1 — Supplementary Material 1 [file 10047_2026_1569_MOESM1_ESM.docx]

**Supplementary Data**

Psychological and Socioeconomic Impact of Long-term Home Management on Patients With Left Ventricular Assist Devices and Their Caregivers: A Nationwide Multicenter Questionnaire Survey

**Supplementary Table S1. Questionnaire items, variables, response formats, and placement in the revised manuscript.**

| **Question** | **Construct** | **Question content / response format or summary** | **Placement in manuscript** |
| --- | --- | --- | --- |
| Q1 | Survey consent / participation agreement | Single answer; agree / do not agree after review of the study information and data-handling statement | Supplementary Table S1 |
| Q2 | Respondent category | Single answer; patient / primary caregiver / other | Methods; Supplementary Table S1 |
| Q3 | Number of caregivers | Patient version: number of caregivers; caregiver version: number of caregivers including the respondent. Choices: 1 / 2 / 3 / 4 / 5 or more / do not know | Table 1; Supplementary Table S2 |
| Q4 | Number of supporters | Patient/caregiver-reported number of supporters. A supporter was defined as a trained person who can assist mainly during emergencies such as pump stoppage. Choices: 1 / 2 / 3 / 4 / 5 or more / none | Table 1; Supplementary Table S2 |
| Q5 | Supporter type | Multiple answers; workplace person, school teacher/staff, friend, relative, visiting nurse, nearby person, other | Supplementary Table S2 |
| Q6 | Sex and age | Sex and age category. Caregiver version also asked the sex/age of the patient being cared for | Table 1; Figure 1 |
| Q7 | Living situation / relationship | Living arrangement and relationship of the main caregiver to the patient / caregiver to the patient | Table 1; Figure 1; Supplementary Table S2 |
| Q8 | Duration since VAD implantation | <6 months; 6 months to <1 year; 1 to <2 years; 2 to <3 years; 3 to <4 years; 4 to <5 years; 5 to <6 years; 6 years or more | Table 1; Supplementary Table S6 |
| Q9 | Time from implantation surgery to first discharge | <3 months / 3 to <6 months / >=6 months | Table 1 |
| Q10 | Indication for VAD | Bridge to transplant (BTT) / destination therapy (DT) / do not know | Table 1; Supplementary Table S8 |
| Q11 | Current work/school status | Multiple answers; commuting to work, working from home, attending school, studying at home/online, other, not working or studying | Table 2; Figure 2; Supplementary Table S3 |
| Q12 | Do you want to work or study now? | For respondents not working/studying: want to, but cannot / do not want to | Table 2; Figure 2; Supplementary Table S3 |
| Q13 | Free-text reason cannot work/study | Narrative comments regarding reasons for inability to work or study | Supplementary Table S3; Supplementary Figure S3 |
| Q14 | Patient reasons for not working/studying or enabling factors | Multiple-response barriers/enablers for patients | Table 2; Figure 2; Supplementary Table S3 |
| Q15 | Caregiver reasons for not working/studying or enabling factors | Multiple-response barriers/enablers for caregivers | Table 2; Figure 2; Supplementary Table S3 |
| Q16 | Daily living independence | Ordered single-choice scale from independent public transportation use to daytime bedbound status | Supplementary Table S5; Supplementary Figure S1 |
| Q17 | Mental state in the past 2 weeks | Two PHQ-2 depressive-symptom items; depressive symptoms defined from the summed score | Table 2; Figure 3; Supplementary Tables S4, S7, and S9 |
| Q18 | Use of services | Current use of visiting nursing, home-helper services, and day-care services | Supplementary Table S5; Supplementary Figure S1 |
| Q19 | VAD handling ability | Appropriately / think can / cannot or concerns / do not know | Table 2; Supplementary Table S5; Supplementary Figure S2 |
| Q20 | Single greatest anxiety about VAD handling | Free-text theme regarding anxiety about VAD handling | Supplementary Figure S2 |
| Q21 | VAD-related emergency experience | No / Yes | Table 2; Supplementary Table S5; Supplementary Figure S2 |
| Q22 | Details of VAD-related emergency experience | Free-text description among respondents with emergency experience | Supplementary Figure S2 |
| Q23 | Burden of 24-hour caregiver accompaniment | Single-answer burden scale; in DT respondents, assessed by recalling the first 6 months after discharge | Table 2; Figure 3; Supplementary Tables S4, S7, S8, and S9 |
| Q24 | Specific content of caregiver-accompaniment burden | Free-text description among respondents reporting burden | Discussion; Supplementary Figure S3 |
| Q25 | Expected impact if 24-hour caregiver accompaniment were no longer required | Free-text expectations regarding changes in daily life, emotions, relationships, and activities | Discussion; Supplementary Figure S3 |
| Q26 | What became possible after VAD implantation | Free-text description of positive changes after VAD implantation | Supplementary Figure S3 |
| Q27 | Currently prohibited / impossible but desired in the future because of VAD | Free-text description of currently restricted activities or rules that respondents wished could be relaxed | Supplementary Figure S3 |

**Supplementary Table S2. Detailed caregiving structure and support network.**

| **Variable** | **Patients (n = 184)** | **Caregivers (n = 136)** |
| --- | --- | --- |
| Number of caregivers |  |  |
| 1 caregiver | 41 (22.3) | 29 (21.3) |
| 2 caregivers | 56 (30.4) | 45 (33.1) |
| 3 caregivers | 44 (23.9) | 33 (24.3) |
| 4 caregivers | 16 (8.7) | 16 (11.8) |
| 5 or more caregivers | 26 (14.1) | 13 (9.6) |
| Unknown | 1 (0.5) | 0 (0.0) |
| Number of supporters |  |  |
| 1 supporter | 28 (15.2) | 15 (11.0) |
| 2 supporters | 29 (15.8) | 29 (21.3) |
| 3 supporters | 28 (15.2) | 28 (20.6) |
| 4 supporters | 14 (7.6) | 9 (6.6) |
| 5 or more supporters | 40 (21.7) | 24 (17.6) |
| No supporter | 45 (24.5) | 31 (22.8) |
| Supporter present (any) | 139 (75.5) | 105 (77.2) |
| Supporter type (multiple response; denominator: all respondents) |  |  |
| Workplace colleague | 38 (20.7) | 24 (17.6) |
| School faculty/staff | 0 (0.0) | 1 (0.7) |
| Friend | 19 (10.3) | 13 (9.6) |
| Relative | 108 (58.7) | 87 (64.0) |
| Visiting nurse | 8 (4.3) | 4 (2.9) |
| Neighbor | 2 (1.1) | 2 (1.5) |
| Other | 8 (4.3) | 7 (5.1) |
| Primary caregiver relationship |  |  |
| Spouse/partner | 135 (73.4) | 102 (75.0) |
| Other than spouse/partner | 49 (26.6) | 34 (25.0) |

*Values are n (%) unless otherwise specified. Supporter type was a multiple-response item.*

**Supplementary Table S3. Detailed work/study status and reasons for inability to work or study.**

| **Variable** | **Patients** | **Caregivers** |
| --- | --- | --- |
| Work/study status (multiple response; denominator: all respondents) |  |  |
| Commuting to workplace | 57 (31.0) | 77 (56.6) |
| Working from home | 32 (17.4) | 12 (8.8) |
| Attending school | 0 (0.0) | 0 (0.0) |
| Distance/online school | 0 (0.0) | 1 (0.7) |
| Other work/study | 22 (12.0) | 7 (5.1) |
| Not working/studying | 81 (44.0) | 43 (31.6) |
| Any work/study | 103 (56.0) | 93 (68.4) |
| Not working/studying | 81 (44.0) | 43 (31.6) |
| Among those not working/studying: wished to work/study but could not | 51/81 (63.0) | 18/43 (41.9) |
| Patient reasons among patients wishing to work/study but unable (n=51; multiple response) |  |  |
| Difficulty obtaining 24-hour accompaniment from the main caregiver | 31/51 (60.8) | - |
| Lack of support from family members other than the main caregiver | 21/51 (41.2) | - |
| Lack of supporter cooperation at workplace/school | 12/51 (23.5) | - |
| Insufficient flexibility at workplace/school | 12/51 (23.5) | - |
| Poor physical condition | 22/51 (43.1) | - |
| Other | 6/51 (11.8) | - |
| Caregiver reasons among caregivers wishing to work/study but unable (n=18; multiple response) |  |  |
| Need to accompany the patient 24 hours a day | - | 12/18 (66.7) |
| Difficulty rotating care with other caregivers | - | 7/18 (38.9) |
| Lack of supporter cooperation at patient's workplace/school | - | 0/18 (0.0) |
| Insufficient flexibility at patient's workplace/school | - | 0/18 (0.0) |
| Insufficient flexibility at own workplace/school | - | 1/18 (5.6) |
| Patient's poor physical condition | - | 1/18 (5.6) |
| Other | - | 4/18 (22.2) |

*Values are n (%) or n/N (%) as indicated. Reasons were multiple-response items.*

**Supplementary Table S4. Psychological burden and PHQ-2 depressive-symptom responses.**

| **Variable** | **Patients (n = 184)** | **Caregivers (n = 136)** |
| --- | --- | --- |
| Caregiver-accompaniment burden (Q23) |  |  |
| Burdened | 108 (58.7) | 38 (27.9) |
| Somewhat burdened | 46 (25.0) | 46 (33.8) |
| Not very burdened | 19 (10.3) | 39 (28.7) |
| Not burdened | 11 (6.0) | 13 (9.6) |
| Burdened or somewhat burdened | 154 (83.7) | 84 (61.8) |
| PHQ-2 item 1: little interest or pleasure |  |  |
| Frequently | 15 (8.2) | 6 (4.4) |
| Sometimes | 41 (22.3) | 33 (24.3) |
| Rarely | 73 (39.7) | 41 (30.1) |
| Not at all | 55 (29.9) | 56 (41.2) |
| PHQ-2 item 2: feeling down, depressed, or hopeless |  |  |
| Frequently | 14 (7.6) | 6 (4.4) |
| Sometimes | 51 (27.7) | 37 (27.2) |
| Rarely | 64 (34.8) | 43 (31.6) |
| Not at all | 55 (29.9) | 50 (36.8) |
| PHQ-2 positivity (score ≥3) | 67 (36.4) | 48 (35.3) |
| PHQ-2 positive among those with caregiver-accompaniment burden | 61/154 (39.6) | 38/84 (45.2) |
| PHQ-2 positive among those without caregiver-accompaniment burden | 6/30 (20.0) | 10/52 (19.2) |

*PHQ-2 positivity was defined as a total score of 3 or higher. Values are n (%) or n/N (%).*

**Supplementary Table S5. Daily living status, home-service use, VAD handling, and emergency experience.**

| **Variable** | **Patients (n = 184)** | **Caregivers (n = 136)** |
| --- | --- | --- |
| Daily living ability (Q16) |  |  |
| Can go out using public transportation or equivalent | 88 (47.8) | 62 (45.6) |
| Can go out within the neighborhood | 33 (17.9) | 24 (17.6) |
| Can go out with assistance and spends most daytime out of bed | 50 (27.2) | 43 (31.6) |
| Rarely goes out and alternates between bed and being up during daytime | 12 (6.5) | 7 (5.1) |
| Mainly stays in bed during daytime but can transfer to wheelchair independently | 1 (0.5) | 0 (0.0) |
| Mainly stays in bed during daytime and can transfer to wheelchair with assistance | 0 (0.0) | 0 (0.0) |
| Needs assistance with toileting, meals, and dressing but can turn over independently | 0 (0.0) | 0 (0.0) |
| Stays in bed during daytime and has difficulty turning over independently | 0 (0.0) | 0 (0.0) |
| Overall use of home services (highest frequency across visiting nursing, home care, and day care) |  |  |
| ≥4 times/week | 4 (2.2) | 2 (1.5) |
| 2–3 times/week | 16 (8.7) | 8 (5.9) |
| Once/week | 8 (4.3) | 13 (9.6) |
| Less often | 6 (3.3) | 4 (2.9) |
| Not using | 150 (81.5) | 109 (80.1) |
| VAD device handling |  |  |
| Can handle appropriately | 85 (46.2) | 51 (37.5) |
| Think can handle | 86 (46.7) | 75 (55.1) |
| Cannot handle/concerns | 9 (4.9) | 9 (6.6) |
| Unknown | 4 (2.2) | 1 (0.7) |
| Any positive response indicating VAD handling was possible | 171 (92.9) | 126 (92.6) |
| VAD-related emergency experience |  |  |
| No VAD-related emergency | 153 (83.2) | 112 (82.4) |
| Experienced a VAD-related emergency | 31 (16.8) | 24 (17.6) |

*Values are n (%). The home-service category represents the highest frequency across visiting nursing, home care, and day-care service items.*

**Supplementary Table S6. LVAD support-duration distribution.**

| **LVAD support duration** | **Patients (n = 184)** | **Caregivers (n = 136)** |
| --- | --- | --- |
| 6 months to <1 year | 9 (4.9) | 10 (7.4) |
| 1 to <2 years | 38 (20.7) | 34 (25.0) |
| 2 to <3 years | 35 (19.0) | 24 (17.6) |
| 3 to <4 years | 38 (20.7) | 23 (16.9) |
| 4 to <5 years | 30 (16.3) | 22 (16.2) |
| 5 to <6 years | 23 (12.5) | 15 (11.0) |
| ≥6 years | 11 (6.0) | 8 (5.9) |

*Percentages are calculated within each respondent group.*

**Supplementary Table S7. Exploratory duration-stratified outcomes.**

| **Group** | **Outcome** | **<1 year** | **1 to <3 years** | **≥3 years** | **p value** |
| --- | --- | --- | --- | --- | --- |
| Patients | Caregiver-accompaniment burden | 7/9 (77.8) | 60/73 (82.2) | 87/102 (85.3) | 0.762 |
| Patients | PHQ-2 positivity | 1/9 (11.1) | 35/73 (47.9) | 31/102 (30.4) | 0.016 |
| Patients | Not working/studying | 4/9 (44.4) | 31/73 (42.5) | 46/102 (45.1) | 0.942 |
| Caregivers | Caregiver-accompaniment burden | 6/10 (60.0) | 37/58 (63.8) | 41/68 (60.3) | 0.916 |
| Caregivers | PHQ-2 positivity | 6/10 (60.0) | 18/58 (31.0) | 24/68 (35.3) | 0.209 |
| Caregivers | Not working/studying | 4/10 (40.0) | 21/58 (36.2) | 18/68 (26.5) | 0.422 |

*p values are from chi-squared tests across the three duration strata. PHQ-2, two-item Patient Health Questionnaire.*

**Supplementary Table S8. Exploratory BTT/DT subgroup outcomes.**

| **Group** | **Outcome** | **BTT** | **DT** | **p value** |
| --- | --- | --- | --- | --- |
| Patients | Caregiver-accompaniment burden | 129/156 (82.7) | 21/24 (87.5) | 0.770 |
| Patients | PHQ-2 positivity | 55/156 (35.3) | 12/24 (50.0) | 0.164 |
| Patients | Not working/studying | 65/156 (41.7) | 13/24 (54.2) | 0.250 |
| Caregivers | Caregiver-accompaniment burden | 68/112 (60.7) | 15/22 (68.2) | 0.510 |
| Caregivers | PHQ-2 positivity | 36/112 (32.1) | 12/22 (54.5) | 0.045 |
| Caregivers | Not working/studying | 34/112 (30.4) | 9/22 (40.9) | 0.332 |

*Respondents with unknown treatment goal were excluded. p values are from chi-squared or Fisher's exact tests as appropriate. BTT, bridge to transplant; DT, destination therapy; PHQ-2, two-item Patient Health Questionnaire.*

**Supplementary Table S9. Multivariable logistic regression for depressive symptoms.**

| **Group** | **Variable** | **Odds ratio (95% CI)** | **p value** |
| --- | --- | --- | --- |
| Patients | Caregiver-accompaniment burden | 2.61 (0.99-6.86) | 0.053 |
| Patients | Not working/studying | 1.51 (0.81-2.80) | 0.196 |
| Patients | LVAD support duration, per year | 0.87 (0.72-1.04) | 0.132 |
| Patients | Number of caregivers | 0.85 (0.67-1.09) | 0.204 |
| Caregivers | Caregiver-accompaniment burden | 3.42 (1.51-7.76) | 0.003 |
| Caregivers | Not working/studying | 1.10 (0.50-2.41) | 0.816 |
| Caregivers | LVAD support duration, per year | 0.91 (0.73-1.13) | 0.392 |
| Caregivers | Number of caregivers | 0.93 (0.69-1.27) | 0.650 |

*Separate multivariable models were fitted for patients and caregivers. Depressive symptoms were defined as PHQ-2 score >=3. CI, confidence interval; LVAD, left ventricular assist device; PHQ-2, two-item Patient Health Questionnaire.*

**Supplementary Figure Legends**

**Supplementary Figure S1. Daily living independence and use of home services.** Panel A summarizes daily living independence. Panel B summarizes home-service use, including visiting nursing, home-helper services, and day-care services.

**Supplementary Figure S2. VAD-handling ability, anxiety themes, and emergency experience.** Panel A summarizes VAD-handling outcomes and emergency experience. Panel B summarizes major free-text anxiety themes related to VAD handling and emergency response.

**Supplementary Figure S3. Qualitative thematic summary of free-text responses.** This figure organizes recurring qualitative themes from free-text responses into work/study restriction, caregiver-accompaniment burden, and desired future changes. The figure is intended as a thematic map rather than a quantitative display.
