# Supplementary figures and images for: Psychological and socioeconomic impact of long-term home management on patients with left ventricular assist devices and their caregivers: a nationwide multicenter questionnaire survey
Source: J Artif Organs. 2026 Jul 9;29(3):43. doi: 10.1007/s10047-026-01569-4 (PMC13350110; doi:10.1007/s10047-026-01569-4)

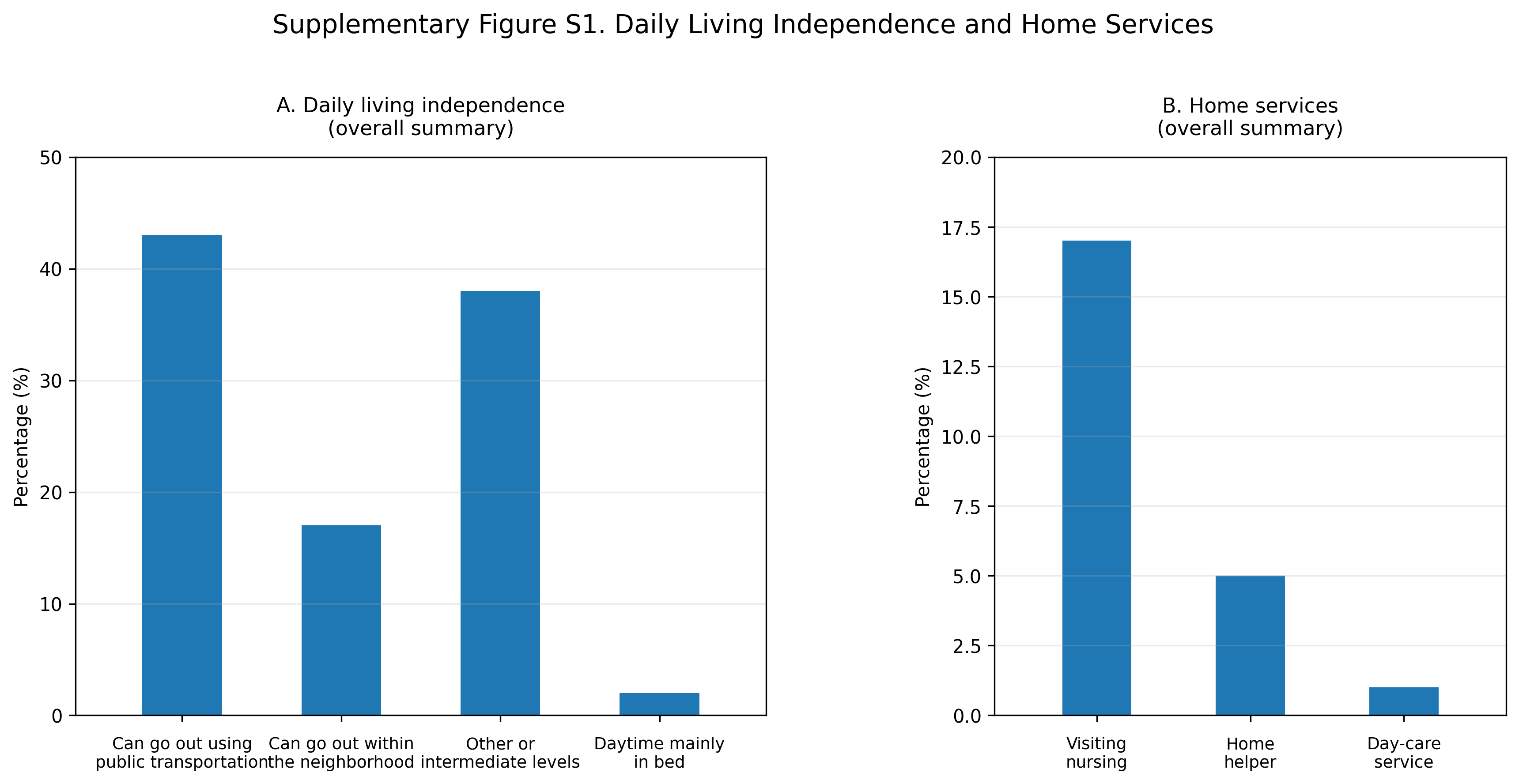

Supplement: Supplementary file 2 — Supplementary Material 1 [file 10047_2026_1569_MOESM2_ESM.png]

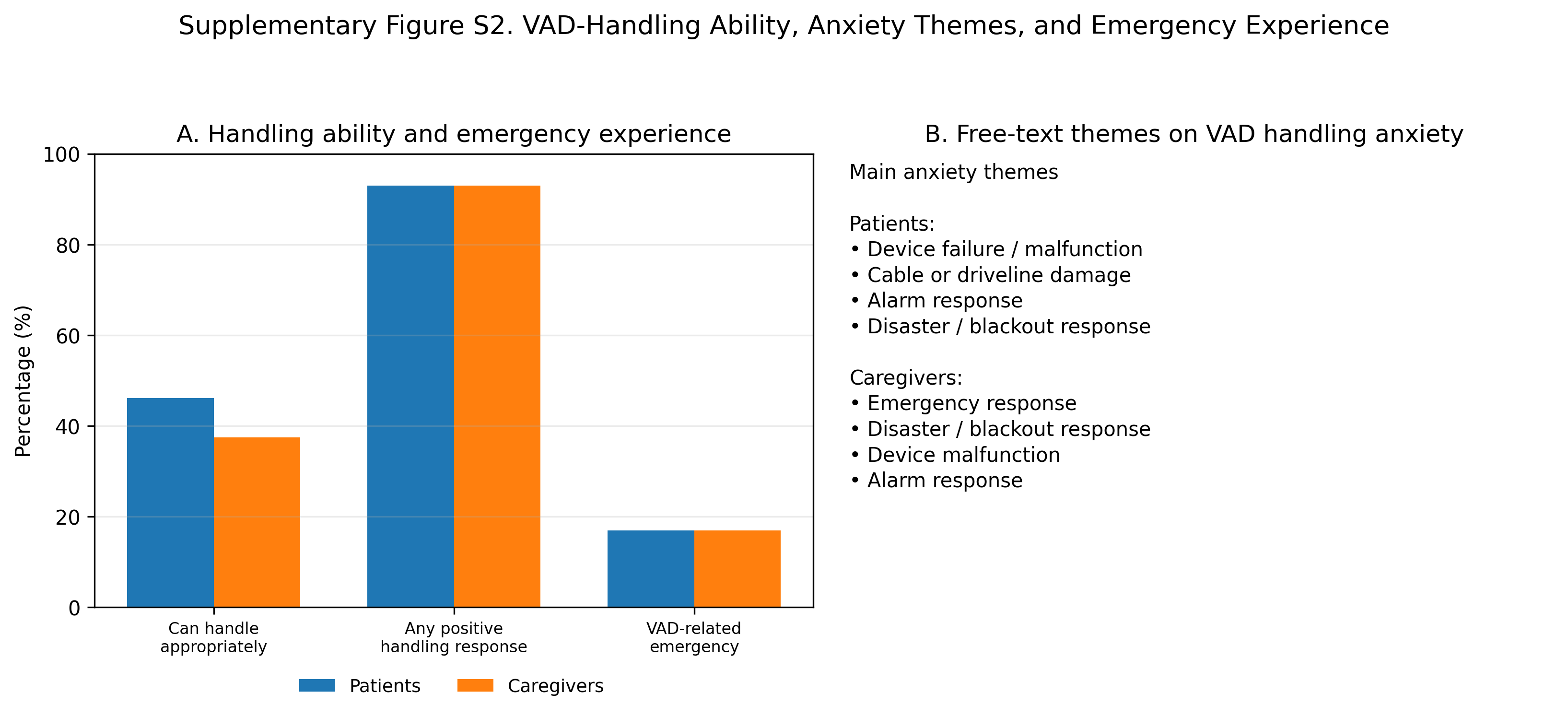

Supplement: Supplementary file 3 — Supplementary Material 2 [file 10047_2026_1569_MOESM3_ESM.png]

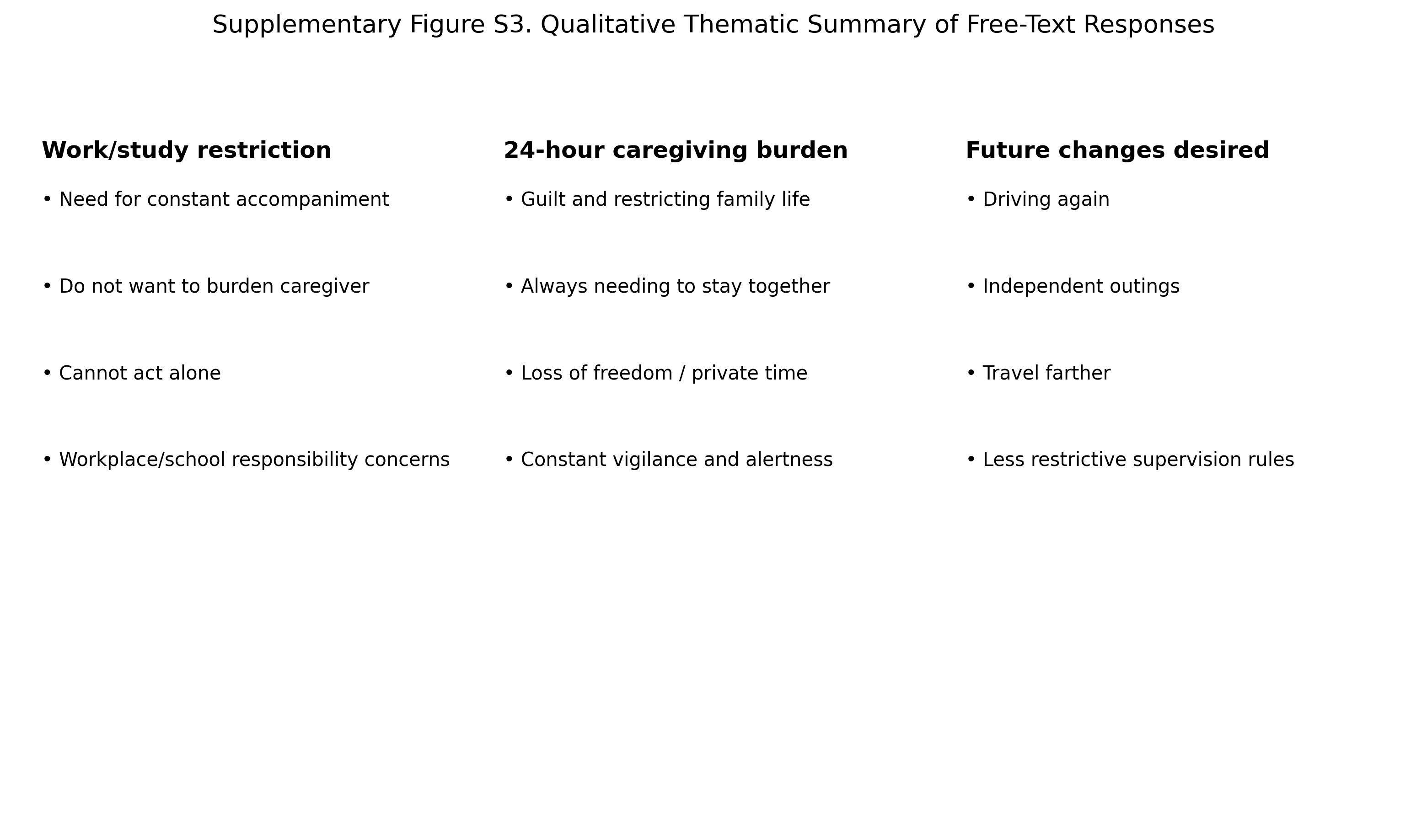

Supplement: Supplementary file 4 — Supplementary Material 3 [file 10047_2026_1569_MOESM4_ESM.png]
